# Supplementary material for: Pre-CAT: A web-based, graphical user-interface toolbox for preclinical CEST-MRI data processing and analysis
Source: ArXiv. 2026 May 1:arXiv:2605.00511v1. Preprint. [Version 1] (PMC13142558)
Supplement: Supplementary file 1 [file NIHPP2605.00511v1-supplement-1.pdf]

SUPPLEMENTAL MATERIAL

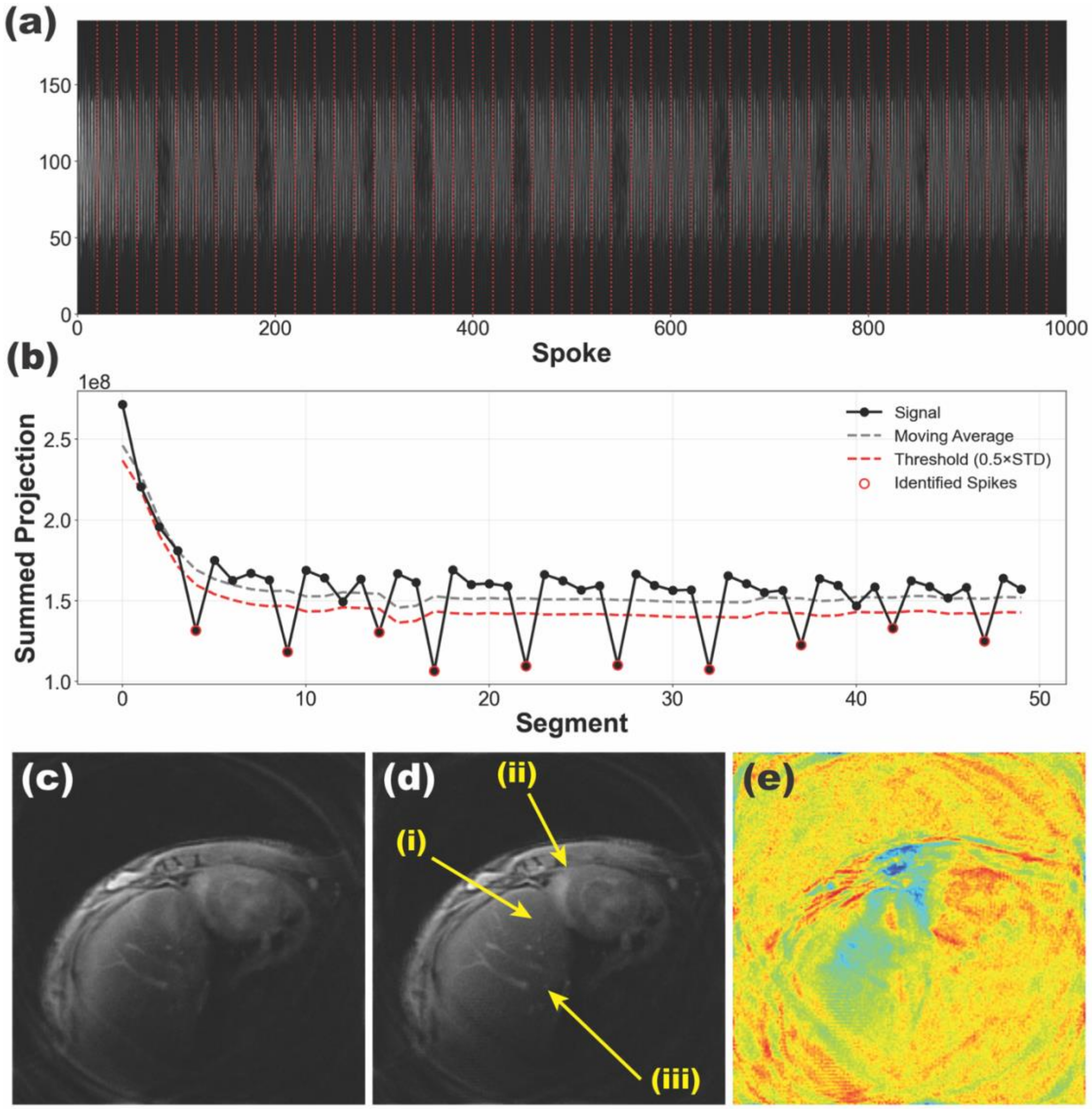

Figure S1: A schematic illustrating the respiratory motion correction workflow. Dark segments in the projection image (a) were identified as periods of respiratory motion. Projections were then summed over each segment, and a moving average was computed to quantitatively identify corrupted segments based on a signal drop threshold. The unfiltered image (c) exhibits blurring around the myocardium and liver. After filtering, the final image (d) demonstrated fewer susceptibility artifacts in the liver (i), improved edge sharpness in the LV and RV myocardium (ii), and enhanced visibility of hepatic vessels (iii). A difference map (raw - filtered) (e) was plotted using the Jet colormap to emphasize affected regions.

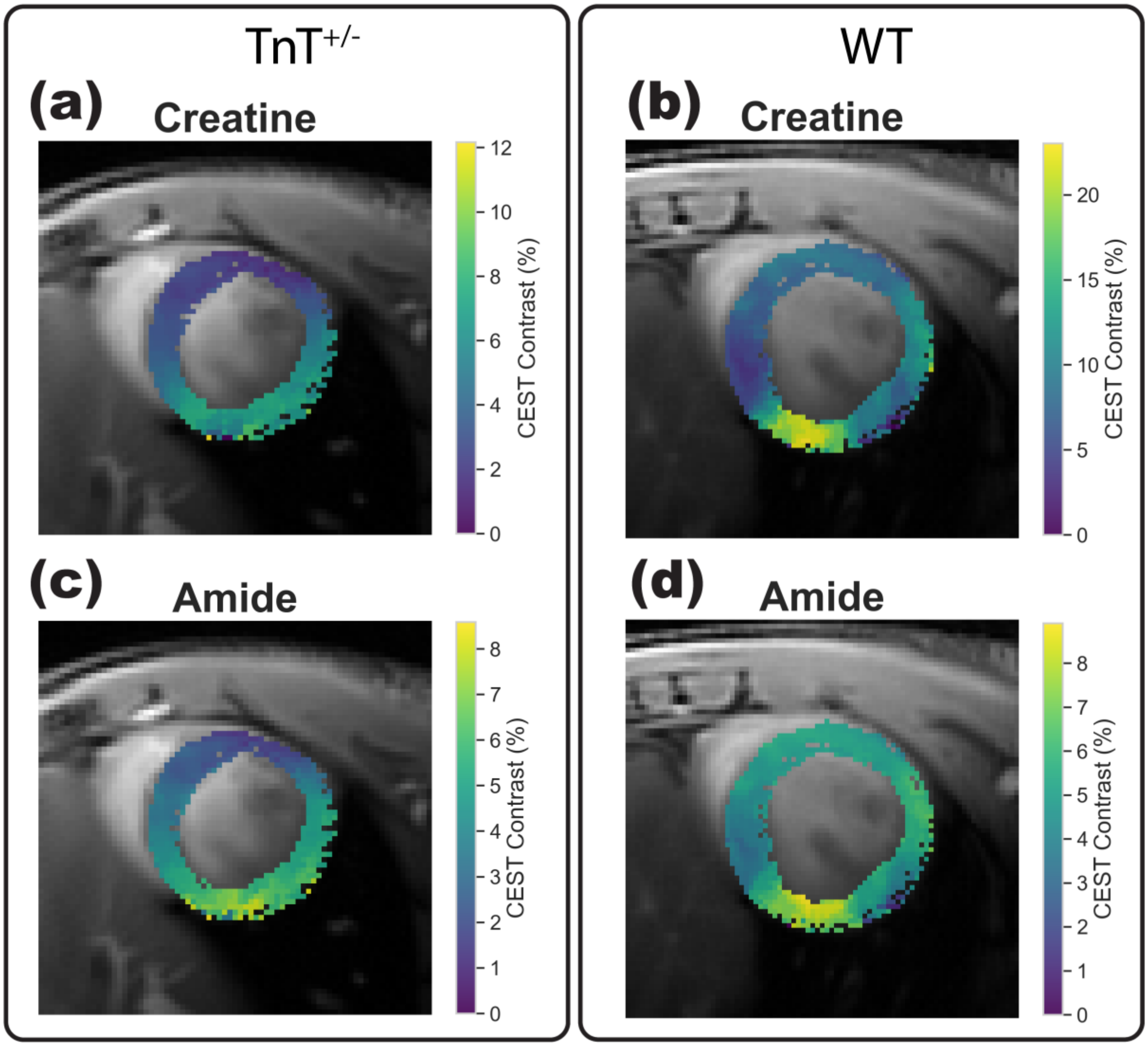

Figure S2: Representative creatine and amide CEST contrast maps in a murine, transgenic model of hypertrophic cardiomyopathy (TnT$^{+/-}$) and a littermate control (WT).

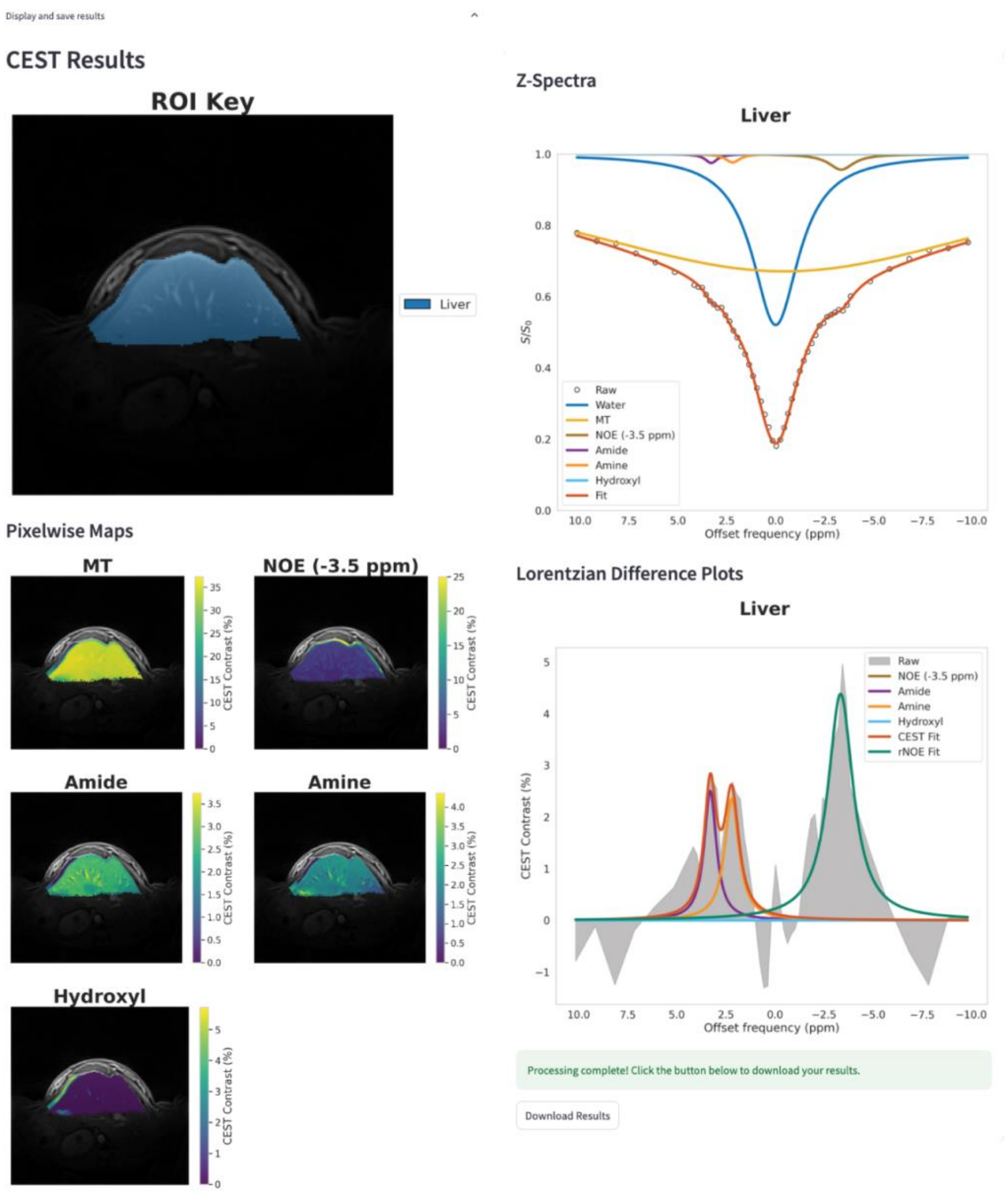

Figure S3: Representative Pre-CAT output data for CEST imaging of the murine liver. Outputs include pixelwise CEST contrast maps and segmentwise Z-spectra with Lorentzian difference plots. This animal was injected with a polyethylene glycol-based hydrogel, as seen in the hydroxyl contrast map.
